# Association of GSTT1, GSTM1, and NQO1 Gene Polymorphisms With Susceptibility, Clinical Severity, and Treatment Response in Aplastic Anemia

**DOI:** 10.7759/cureus.90930

**Published:** 2025-08-25

**Authors:** Vijayta Singh, Anam Ahmad, Alka Diwedi, Mayank Jain, Anil Kumar Tripathi, Saurabh Shukla

**Affiliations:** 1 Pathology, Maharaja Suhel Dev Autonomous State Medical College and Mahrishi Balark Hospitals, Bahraich, IND; 2 Anatomy, Era's Lucknow Medical College and Hospital, Lucknow, IND; 3 Clinical Hematology, King George's Medical University, Lucknow, IND; 4 Thoracic Surgery, King George's Medical University, Lucknow, IND; 5 Hematology, King George's Medical University, Lucknow, IND

**Keywords:** aplastic anemia, detoxification enzymes, gstm1, gstt1, nqo1, oxidative stress, polymorphism

## Abstract

Introduction

Aplastic anemia (AA) is an uncommon hematologic condition in which markedly reduced bone marrow cellularity results in decreased production of red blood cells, white blood cells, and platelets. Its etiology is complex and multifactorial, with evidence suggesting an interplay of immune-mediated destruction of hematopoietic progenitor cells, environmental exposures, and genetic predisposition. In this study, we focused on genetic polymorphisms in detoxification enzymes, which may influence individual susceptibility to AA by altering the ability to manage oxidative stress and metabolize xenobiotics.

Methods

We enrolled patients with AA (n = 200) and age-matched healthy controls (n = 200). Genotyping of GSTM1 and GSTT1 (null/present polymorphisms) was performed using multiplex PCR, while the NQO1 C609T polymorphism (rs1800566) was determined by PCR-restriction fragment length polymorphism. Genotype distributions were compared using chi-square tests, and ORs with 95% CIs were calculated.

Results

The GSTT1 null genotype and NQO1 CT genotype were significantly associated with increased susceptibility to AA (p < 0.05). The GSTT1 null polymorphism was also correlated with both severe and non-severe disease according to the Camitta criteria. GSTM1 showed a trend toward association but was not statistically significant with respect to treatment response (p > 0.05).

Conclusions

The GSTT1 null and NQO1 CT genotypes are associated with increased susceptibility to AA. In addition, the GSTT1 null genotype predicts poor response to immunosuppressive therapy, indicating its potential as a prognostic biomarker.

## Introduction

Aplastic anemia (AA) is a rare but potentially fatal hematologic disorder characterized by peripheral blood pancytopenia and bone marrow hypocellularity [1,2]. First described in the late 19th century, its incidence ranges from 1.4 to 14 cases per million population [3]. The pathogenesis of AA primarily involves immune-mediated destruction of hematopoietic progenitors, while environmental and genetic factors also play significant roles in disease development [4]. In recent years, the contribution of genetic susceptibility has gained increasing attention [5-7]. Notably, genetic polymorphisms in detoxification enzymes such as glutathione S-transferases (GSTs) and NAD(P)H: quinone oxidoreductase 1 (NQO1) have been investigated for their potential role in modulating individual risk for various malignancies and bone marrow failure syndromes [8-10]. These enzymes are critical in cellular defense against oxidative damage and in the metabolism of toxic electrophilic compounds, including benzene derivatives, which are recognized environmental risk factors for AA.

GST theta 1 (GSTT1) and GST mu 1 (GSTM1), members of the GST supergene family, detoxify reactive oxygen species and harmful xenobiotics [11]. Homozygous deletions in these genes, known as null genotypes, result in complete loss of enzymatic activity, thereby compromising cellular detoxification mechanisms [12,13]. Similarly, the NQO1 C609T polymorphism causes a proline-to-serine substitution at codon 187, which impairs enzyme stability and activity. Individuals homozygous for the T allele (TT) completely lack functional NQO1 protein [14]. Beyond influencing disease susceptibility, these polymorphisms may also affect the severity of AA and patient response to immunosuppressive therapy (IST). Evidence suggests that impaired detoxification may increase the vulnerability of hematopoietic cells to immune-mediated destruction or environmental insults, thereby impacting prognosis [15,16].

This study aims to investigate the relationship between polymorphisms in GSTT1, GSTM1, and NQO1 genes with susceptibility to AA, its clinical severity, and response to IST. A better understanding of these associations may improve risk stratification, facilitate early diagnosis, and support the development of personalized therapeutic approaches.

## Materials and methods

Study design and participants

This prospective case-control study was conducted between 2013 and 2016 at the Department of Hematology, King George’s Medical University, Lucknow, India. A total of 200 patients diagnosed with AA and 200 age- and sex-matched healthy controls were enrolled. Healthy controls were recruited from among the biological relatives of patients. Patients were categorized into non-severe (NSAA), severe (SAA), and very severe (VSAA) groups according to modified criteria described by Bacigalupo et al. and Marsh et al. [17,18]. Control participants had no history of hematological malignancies, autoimmune disorders, or recent exposure to cytotoxic agents. Written informed consent was obtained from all participants, and the study was approved by the Institutional Ethics Committee in accordance with the Declaration of Helsinki.

Clinical classification and treatment response

Patients were classified according to the Modified Camitta Criteria, which define bone marrow cellularity as ≤25% (or 25-50% with <30% residual hematopoietic cells). NSAA was defined by hypocellular marrow with cytopenia not fulfilling criteria for severe disease. SAA was diagnosed if two or more of the following were present: absolute neutrophil count (ANC) <500/μL, platelet count <20,000/μL, or reticulocyte count <60,000/μL. VSAA was diagnosed using the same criteria as SAA, but with an ANC <200/μL. Response to IST was evaluated six months after treatment. Patients achieving complete or partial hematologic recovery were considered responders, while those with persistent pancytopenia were classified as nonresponders.

Sample collection and DNA extraction

Peripheral blood (5 mL) was collected in EDTA vials from all participants. Genomic DNA was extracted using the Qiagen DNA extraction kit (Qiagen, Hilden, Germany) according to the manufacturer’s protocol. DNA quality and concentration were assessed by 1% agarose gel electrophoresis and NanoDrop spectrophotometry (Thermo Fisher Scientific Inc., Waltham, MA, USA).

Genotyping procedure

Genotyping for GSTT1 and GSTM1 gene deletions was performed using multiplex PCR, while the NQO1 C609T polymorphism was analyzed by PCR-restriction fragment length polymorphism. Amplification of the β-globin gene served as an internal control. For GSTM1, a 215 bp band indicated presence, while absence indicated a null genotype. For GSTT1, a 459 bp band indicated presence, while absence indicated a null genotype. The NQO1 (rs1800566) gene was amplified as a 524 bp product and digested with HinfI. Digestion produced 188 bp and 85 bp fragments for the CC genotype; 524 bp, 188 bp, and 85 bp fragments for the CT genotype; and 524 bp, 151 bp, and 85 bp fragments for the TT genotype. Primer sequences, PCR product sizes, and additional genotyping details are provided in Table 1.

**Table 1:** Primer sequences, PCR product sizes, and genotyping details RFLP, restriction fragment length polymorphism

Gene	Polymorphism	Primer sequence (5′-3′)	PCR product size	Genotyping method	Digestive enzyme	Fragment sizes (bp)
GSTM1	Null/present	F: GAACTCCCTGAAAAGCTAAAGC R: GTTGGGCTCAAATATACGGTGG	215 bp	Multiplex PCR	None	215 bp (present), no band (null)
GSTT1	Null/present	F: TCACCGGATCATGGCCAGCA R: TTCCTTACTGGTCCTCACATCTC	459 bp	Multiplex PCR	None	459 bp (present), no band (null)
NQO1	C609T	F: AGTGGCATTCTGCATTTCTGTG R: GATGGACTTGCCCAAGTGATG	524 bp	PCR-RFLP	HinfI	CC: 524 bp CT: 524 bp, 188 bp, 151 bp, 85 bp TT: 188 bp, 151 bp, 85 bp

PCR conditions and gel electrophoresis

PCR reactions were performed in a 25 μL volume containing 100 ng of genomic DNA, 10 pmol of each primer, 200 μM dNTPs, 1.5 mM MgCl₂, and 1 unit of Taq DNA polymerase. The cycling protocol consisted of an initial denaturation at 95°C for five minutes, followed by 35 cycles of denaturation at 95°C for 30 seconds, annealing at 60°C for 45 seconds, and extension at 72°C for 45 seconds, with a final extension at 72°C for seven minutes. PCR products were resolved by agarose gel electrophoresis: 2% gels for GSTT1 and GSTM1 and 3% gels for digested NQO1 fragments. Gels were stained with ethidium bromide and visualized under UV illumination.

Statistical analysis

Allelic and genotypic frequencies were determined by direct counting. The Hardy-Weinberg equilibrium for the NQO1 gene was assessed in the control group to confirm genetic stability. Differences between patient and control groups were analyzed using the chi-square test or Fisher’s exact test, as appropriate. ORs with 95% CIs were calculated through logistic regression to estimate the strength of associations. All statistical analyses were conducted using IBM SPSS Statistics for Windows, Version 25.0 (Released 2017; IBM Corp., Armonk, NY, USA). A p-value <0.05 was considered statistically significant.

## Results

The distribution of polymorphisms in the GSTT1, GSTM1, and NQO1 genes was analyzed among AA patients (n = 200) and healthy controls (n = 200) to assess whether the presence or absence of functional alleles of these detoxification-related genes correlates with disease susceptibility, clinical severity, or response to IST. Genotype frequencies were further stratified and analyzed across patient subgroups, including severity categories (NSAA, SAA, and VSAA) and treatment outcomes (responders vs. nonresponders). The demographic variables and clinical characteristics of AA patients and healthy controls are summarized in Table 2.

**Table 2:** Demographic details of patients with acquired AA and healthy control subjects AA, aplastic anemia; IST, immunosuppressive therapy; NSAA, non-severe aplastic anemia; SAA, severe aplastic anemia; VSAA, very severe aplastic anemia

Characteristic	Patients (N = 200)	Controls (N = 200)
Age (mean ± SD)	29.13 ± 16.4	27.92 ± 8.9
Sex		
Male (%)	138 (69.0%)	137 (68.5%)
Female (%)	62 (31.0%)	63 (31.5%)
Patients’ classification based on disease severity		
SAA (%)	88 (44%)	0 (0)
NSAA (%)	94 (47%)	0 (0)
VSAA (%)	18 (9%)	0 (0)
Response to IST		
Responders (complete + partial) (%)	105 (52.5%)	0 (0)
Nonresponders (%)	95 (47.5%)	0 (0)

Table 3 presents the frequencies of GSTM1, GSTT1, and NQO1 genotypes among AA patients and healthy controls, along with the corresponding ORs and CIs. The GSTT1 null genotype was significantly associated with susceptibility to AA (OR 1.71, 95% CI: 1.14-2.58, p = 0.0131), indicating that individuals lacking the GSTT1 gene have a higher risk of developing AA. Similarly, the NQO1 CT heterozygous genotype was significantly associated with disease susceptibility (OR 2.60, 95% CI: 1.28-5.28, p = 0.0106). The GSTM1 null genotype, as well as the NQO1 TT genotype and T allele, were more frequent in patients than in controls; however, these associations were not statistically significant. These findings suggest that the GSTT1 null and NQO1 CT genotypes may serve as genetic risk factors for AA.

**Table 3:** Influence of GSTT1, GSTM1, and NQO1 polymorphisms on susceptibility to AA in patients and control subjects ^*^ Statistically significant susceptible genotype; p < 0.05 was considered statistically significant. AA, aplastic anemia

Gene polymorphism	Controls (%) (n = 200)	Patients (%) (n = 200)	p-Value	OR (95% CI)
GSTM1 genotype				
Present	128 (64%)	110 (55%)	-	Reference
Null	72 (36%)	90 (45%)	0.0834	1.45 (0.97-2.17)
GSTT1 genotype				
Present	138 (69%)	113 (56.5%)	-	Reference
Null	62 (31%)	87 (43.5%)	0.0131^*^	1.71 (1.14-2.58)
NQO1 (C/T) genotype				
CC (wild type)	183 (91.5%)	164 (82%)	-	Reference
CT (heterozygous)	12 (6%)	28 (14%)	0.0106^*^	2.60 (1.28-5.28)
TT (mutant)	5 (2.5%)	8 (4%)	0.466	1.78 (0.57-5.54)
Allele frequency				
C	368 (92%)	356 (89%)	-	Reference
T	32 (8%)	44 (11%)	0.1847	1.45 (0.97-2.17)

Table 4 evaluates the association between GSTM1, GSTT1, and NQO1 genotypes and different clinical severity groups of AA compared with healthy controls. The GSTM1 null genotype was significantly associated only with the NSAA group (OR 1.78, 95% CI: 1.08-2.92, p = 0.0301), suggesting a potential role in the development of less severe disease. The GSTT1 null genotype was significantly associated with both the SAA (OR 1.94, 95% CI: 1.16-3.25, p = 0.016) and NSAA (OR 1.80, 95% CI: 1.08-2.98, p = 0.0263) groups, indicating that this variant may increase the risk of disease across a spectrum of severities. Notably, the NQO1 mutant genotype (CT+TT) demonstrated a strong and statistically significant association with the VSAA AA subgroup (OR 8.61, 95% CI: 3.00-24.71, p = 0.0002), suggesting that NQO1 variation may serve as a marker of particularly aggressive disease. No significant associations were found for the GSTT1 null genotype in VSAA AA or for NQO1 mutants in other subgroups.

**Table 4:** Influence of GSTT1, GSTM1, and NQO1 polymorphisms on SAA and NSAA patients and control subjects SAA, NSAA, and VSAA refer to clinical categories of AA. p < 0.05 was considered statistically significant; an asterisk (^*^) denotes statistical significance. AA, aplastic anemia; NSAA, non-severe aplastic anemia; SAA, severe aplastic anemia; VSAA, very severe aplastic anemia

Genotype	SAA (n = 88)	NSAA (n = 94)	VSAA (n = 18)	Controls (n = 200)	SAA vs. Controls OR (95% CI), p-Value	NSAA vs. Controls OR (95% CI), p-Value	VSAA vs. Controls OR (95% CI), p-Value
GSTM1							
Present	53 (60.2%)	47 (50.0%)	10 (55.6%)	128 (64.0%)	Reference	Reference	Reference
Null	35 (39.8%)	47 (50.0%)	8 (44.4%)	72 (36.0%)	1.17 (0.70-1.97), p = 0.5969	1.78 (1.08-2.92), p = 0.0301^*^	1.42 (0.54-3.76), p = 0.6105
GSTT1							
Present	47 (53.4%)	52 (55.3%)	14 (77.8%)	138 (69.0%)	Reference	Reference	Reference
Null	41 (46.6%)	42 (44.7%)	4 (22.2%)	62 (31.0%)	1.94 (1.16-3.25), p = 0.016^*^	1.80 (1.08-2.98), p = 0.0263^*^	0.64 (0.20-2.01), p = 0.5947
NQO1 (CT + TT)							
Mutant (CT + TT)	13 (14.8%)	15 (15.9%)	8 (44.4%)	17 (8.5%)	1.87 (0.86-4.03), p = 0.1414	2.04 (0.97-4.30), p = 0.0702	8.61 (3.00-24.71), p = 0.0002^*^

Table 5 presents the relationship between GSTT1, GSTM1, and NQO1 genotypes and response to IST among AA patients. The GSTT1 null genotype was significantly less frequent among responders compared with nonresponders (OR 0.45, 95% CI: 0.25-0.79, p = 0.0068), suggesting that the absence of the GSTT1 gene is associated with a reduced likelihood of response to therapy. Neither the GSTM1 null genotype nor the NQO1 mutant genotypes showed a statistically significant association with treatment response. These findings indicate the potential utility of GSTT1 genotyping as a predictor of therapeutic outcome in AA patients.

**Table 5:** GSTT1, GSTM1, and NQO1 genotype distribution in responders (complete + partial) and nonresponders ^*^ Statistically significant susceptible genotype

Gene polymorphism	Responders (n = 105)	Nonresponders (n = 95)	p-Value	OR (95% CI)
GSTT1 genotype				
Present	48 (45.7%)	62 (65.3%)	-	Reference
Null	57 (54.3%)	33 (34.7%)	0.0068^*^	0.45 (0.25-0.79)
GSTM1 genotype				
Present	54 (51.4%)	59 (62.1%)	-	Reference
Null	51 (48.6%)	36 (37.9%)	0.1536	0.65 (0.37-1.14)
NQO1 (CT + TT)				
CC (wild type)	84 (80.0%)	80 (84.2%)	-	Reference
CT + TT	21 (20.0%)	15 (15.8%)	0.4665	0.75 (0.36-1.56)
NQO1 allele frequency				
C	184 (88.0%)	172 (90.5%)	-	Reference
T	26 (12.0%)	18 (9.5%)	0.4243	0.74 (0.39-1.40)

## Discussion

This study provides compelling evidence that genetic polymorphisms in detoxification enzymes, particularly GSTT1 and NQO1, are significant determinants of susceptibility and clinical variability in AA among Indian patients. Our findings support and expand upon previous work showing that the GSTT1 null genotype is more prevalent among AA cases than controls, implicating a deficiency in GST-mediated detoxification as a central mechanism underlying marrow failure [2,13,19,20]. The higher frequency of GSTT1 and GSTM1 null genotypes observed in our cohort is consistent with data from both Indian and East Asian populations, suggesting that these genetic risk factors may have broad relevance across diverse ethnic backgrounds [2,20].

The role of the NQO1 C609T polymorphism in AA risk is also underscored by our data. The increased presence of the CT and TT genotypes among patients, particularly those with severe disease, highlights the importance of NQO1 in cellular antioxidant defense. Similar associations have been reported in studies from Europe and Asia, showing that NQO1 deficiency increases susceptibility to oxidative stress and may contribute to DNA damage in hematopoietic stem cells, thereby predisposing individuals to AA [10,14,21]. Our observation of a strong association between the NQO1 mutant genotype and VSAA aligns with evidence that impaired redox regulation exacerbates disease severity, a pattern corroborated by other reports linking oxidative stress markers and genetic vulnerability in marrow failure [3].

Beyond GST and NQO1, recent research has explored the contribution of cytokine and DNA repair gene polymorphisms to AA pathogenesis. Our study complements prior work demonstrating that variants in pro-inflammatory cytokine genes, such as TNF-α and IL-1β, modulate disease risk and prognosis, likely through effects on immune-mediated destruction of progenitor cells and the bone marrow microenvironment [22,23]. Shukla et al. reported that TNF-α-308 and IFN-γ-874 polymorphisms, as well as plasma cytokine levels, were associated with disease severity and response to therapy, reinforcing the multifactorial nature of AA pathogenesis in Indian populations [1,2]. Additionally, studies of DNA repair genes such as XRCC1 have shown that certain variants may enhance susceptibility to AA, particularly when combined with GST or NQO1 deficiency, highlighting the complex interplay between environmental toxins, genetic background, and the cellular machinery responsible for maintaining genomic integrity [19,21].

Notably, the present findings regarding response to IST further illustrate the clinical importance of these genetic markers. Our data reveal that the GSTT1 null genotype was significantly less frequent among responders, suggesting that impaired detoxification may hinder marrow recovery following IST. This observation echoes previous reports from both Indian and international cohorts, supporting the potential utility of GSTT1 and related polymorphisms as predictive biomarkers for therapeutic response [24,25]. In contrast, neither the GSTM1 nor NQO1 genotypes showed statistically significant associations with IST response in our study, which is consistent with mixed results in the literature and suggests that other genetic or environmental factors may influence treatment outcomes [4].

The clinical implications of these results are substantial. Genetic screening for GSTT1 and NQO1 variants, as well as cytokine polymorphisms, could improve risk stratification and inform personalized management of AA, particularly in populations with high environmental or occupational toxin exposure [3,9]. For high-risk genotypes, early intervention, closer monitoring, or alternative therapies (such as hematopoietic stem cell transplantation) might be considered. Additionally, integrating genetic, immunological, and environmental data is likely to further refine prognostic models, as supported by recent meta-analyses and multicenter studies [21].

Despite its strengths, our study has several limitations. The single-center design and modest sample size for certain subgroups, particularly VSAA, may limit the generalizability of our findings. Furthermore, we did not directly assess environmental exposures (such as benzene), which may act synergistically with genetic risk factors. Previous reports have emphasized the importance of such gene-environment interactions in AA pathogenesis, especially in regions with substantial industrial or agricultural chemical exposure [1,21]. Future research should focus on larger, multiethnic cohorts, comprehensive genetic and environmental assessments, and functional studies to elucidate the precise mechanisms underlying these associations [16,26].

## Conclusions

This study highlights that the GSTT1 null and NQO1 CT genotypes are significantly associated with increased susceptibility to AA. The NQO1 CT+TT genotype also correlates with very severe disease, indicating its potential as a marker of disease severity. Additionally, the GSTT1 null genotype was linked to poor response to IST, suggesting its predictive value in treatment outcomes. These findings underscore the role of detoxification gene polymorphisms in AA pathogenesis and prognosis.
